# Differential expression of interferon-induced microRNAs in patients with chronic hepatitis C virus infection treated with pegylated interferon alpha

**DOI:** 10.1186/1743-422X-7-311

**Published:** 2010-11-12

**Authors:** Carolina Scagnolari, Pompea Zingariello, Jacopo Vecchiet, Carla Selvaggi, Delia Racciatti, Gloria Taliani, Elisabetta Riva, Eligio Pizzigallo, Guido Antonelli

**Affiliations:** 1 Department of Molecular Medicine, Laboratory of Virology, "Sapienza" University of Rome; Rome, Italy; 2 Department of Medicine and Science of Aging, Infectious Disease Clinic, G. d'Annunzio University, School of Medicine, Chieti, Italy; 3 Department of Infectious and Tropical Diseases, "Sapienza" University of Rome, Rome, Italy; 4 Virology section, University Campus Bio-Medico, Rome, Italy

## Abstract

There have been reports of in-vitro interferon (IFN)-mediated antiviral activity against the hepatitis C virus (HCV) through microRNAs (miRNAs). The main aim of this study was to evaluate the expression of several miRNAs (miR-1, miR-30, miR-128, miR-196, miR-296) in peripheral blood mononuclear cells (PBMCs) from healthy individuals after in vitro IFN-treatment and in PBMCs from patients with chronic hepatitis C (CHC) before and 12 hours after the first injection of pegylated IFN alpha. We demonstrated that expression of these miRNAs could be recorded in PBMCs collected from healthy individuals before and after in-vitro IFN alpha treatment. Our analysis revealed that the levels of expression of all miRNAs investigated in patients with CHC were different to those in healthy individuals. When levels of the miRNAs were measured 12 hours after the first IFN injection, increases in expression levels of IFN-induced miRNAs were observed in 25-50% of patients, depending on the type of miRNA examined. No correlations were observed between HCV viral load, alanine aminotransferase status and expression of miRNA. Together these findings suggest that: (i) IFN alpha in-vitro treatment of PBMCs leads to a transcriptional induction of all miRNAs investigated; (ii) miRNAs can be induced differentially by IFN treatment in patients with HCV. Given the importance of miRNAs in defending the host against virus infections, it is possible that IFN-induced miRNAs may represent an important determinant of the clinical outcome of IFN therapy in HCV infection.

## Introduction

MicroRNAs (miRNAs) are an important class of small non-coding RNA molecules that have recently come to prominence as critical regulators in a wide array of mechanisms of cell physiology. There is increasing evidence that miRNAs may also have an important function in viral replication and may be used by host cells to control viral infection [1,2]. Indeed, it has been demonstrated that viral RNAs and the miRNA machinery may interact in various ways. First, mammalian viruses encode miRNAs that can act on both the control of viral genes and of cellular genes by repressing their expression. Second, cellular miRNAs may recognize viral RNAs and silence them, or control the expression of a cellular protein necessary for the virus life cycle.

It has also been suggested that miRNAs may be an effector in the classical vertebrate innate immune system [3], and recently an even more direct link between IFN and miRNAs has emerged [4]. Interferon (IFN) beta has been reported as modulating the expression of several cellular miRNAs that are capable of inhibiting hepatitis C virus (HCV) replication and infection, because they have sequence-predicted targets within the HCV genomic RNA. In addition, Pederson and co-authors reported that IFN beta downregulated the expression of miR-122, which has been implicated in the control of HCV RNA replication. This finding could lead to a better understanding of the factors involved in the failure of IFN therapy in patients with chronic hepatitis C (CHC). Due to different viral, environmental and host factors, a sustained virological response is achieved in about 50% of patients infected with HCV genotype 1 and in about 80% of patients infected with HCV genotypes 2 or 3; more importantly, despite extensive examination of the biological and clinical effects of IFN in patients with CHC, the prediction of treatment responses in individual patients still remains difficult [5,6].

In the framework of a study aimed at further characterizing the state of responder, and at improving our knowledge and understanding of IFN therapy effects on patients with CHC, we undertook in-vitro and ex-vivo expression analyses of cellular miRNAs that had previously been reported as being involved in IFN-mediated antiviral activity against HCV [4], using real-time quantitative reverse transcription polymerase chain reaction (RT-PCR) assay. The *ex-vivo *analysis was undertaken before and 12 hours after the first injection of pegylated IFN alpha in CHC patients. Gene expression analysis of MxA, a well-characterized IFN type I gene, was also undertaken as a control. The association between miRNA expression and alanine aminotransferase (ALT) status, HCV genotype, HCV-RNA and response to therapy was evaluated.

## Methods

### Patients and healthy blood donors

Peripheral blood samples were obtained from 12 patients with hepatitis C and ten healthy volunteers. The patients with HCV were treated by subcutaneous injection with either 180 μg PegIFN alpha-2a (PEGASYS; Hoffmann-LaRoche, Basel, Switzerland) (n = 9) or 1.5 μg/kg PegIFN alpha-2b (PegIntron; Schering-Plough, Kenilworth, NJ, USA) (n = 3) plus ribavirin. Treatment duration was 24 or 48 weeks according to HCV genotype. Patients who were HCV-RNA negative after 24 weeks of post-treatment follow-up were considered sustained viral responders. The demographic and clinical data of patients at the time of sample collection are summarized in Table 1. None of the patients had been treated previously with IFNs or other immunosuppressive therapy (treatment-naïve patients). Written informed consent was obtained from each patient, and the study was approved by the Ethics Committees and/or Institutional Review Boards of the participating institutions. PBMCs from healthy donors were treated with 100 international unit (IU)/ml of IFN alpha [leukocyte, Alfaferone (AlfaWassermann, Bologna, Italy)] for 20 hours, the incubation time selected in previous studies aimed at the measurement of IFN-stimulated genes (ISGs) [7,8].

**Table 1:** Demographic and clinical characteristics of patients with chronic hepatitis C

Patient no.	Sex	Age	HCV GT*	Baseline HCV-RNA (copies/ml)	4 weeks HCV-RNA (copies/ml)	4-weeks response*	12 weeks HCV-RNA (copies/ml)	12-weeks response	Ribavirin (mg)	Weight, (Kg)	Type of peg IFN	AST	ALT	FOLLOW-UP
1	F	62	2a/2c	8224917	NEG	R	NEG	R	800	70	Alfa 2a	28	31	SVR
2	F	69	2	19948356	NEG	R	NEG	R	1000	78	Alfa 2a	28	30	SVR
3	F	39	2c	2748250	NEG	R	NEG	R	800	64	alfa 2a	30	38	SVR
4	F	51	2a/2c	4486254	NEG	R	NEG	R	1000	68	Alfa 2a	17	24	SVR
5	M	47	1b	1573026	6800	NR	NEG	R	1200	82	Alfa 2a	49	68	SVR
6	M	67	1a	6018477	21700	NR	NEG	R	1000	78	Alfa 2a	137	214	SVR
7	F	68	1b	941010	NEG	R	NEG	R	1000	74	Alfa 2a	25	54	SVR
8	M	47	2a/2c	3347068	445000	NR	937560	NR	1000	80	Alfa 2b	93	172	NR
9	M	49	2a	2859813	437000	NR	378000	NR	1000	84	Alfa 2b	110	115	NR
10	F	44	1a	2505703	17500	NR	327000	NR	1200	68	Alfa 2a	23	26	NR
11	F	66	1b	21967894	1200000	NR	2550	NR	800	51	Alfa 2a	44	80	NR
12	F	42	1a	1469947	161000	NR	16100	NR	1000	72	Alfa 2b	73	110	NR

*GT, genotype; R, responder, SVR, sustained viral responder; NR, non-responder; IFN, interferon; HCV, hepatitis C virus; AST, aspartate aminotransferase; ALT, alanine aminotransferase.

PBMCs from CHC patients were collected at baseline and 12 hours after the first injection of pegylated IFN alpha. The timing was determined by the following: first, only two sample collections (i.e., pre- and post-dose) were considered to be suitable by the Ethics Committee; second, previous reports had shown significant changes in ISGs expression 12 hours after IFN type I administration in patients with different chronic diseases, including CHC [9-14].

### Blood sampling

Venous peripheral blood from each patient and healthy control was drawn into tubes containing ethylenediaminetetraacetic acid. Peripheral blood mononuclear cells (PBMCs) were separated using Ficoll-Hypaque gradient sedimentation; 5 × 10^6 ^PBMCs were collected, pelleted and frozen at -80°C until examined. After centrifuging, plasma samples were stored at -80°C until required.

### Taqman quantitative RT-PCR for MxA-mRNA

MxA gene transcripts in PBMCs from patients with CHC and healthy individuals were quantified by a real time 5' exonuclease RT-PCR Taqman assay using an ABI 7000 sequence detector (Applied Biosystems, Monza, Italy). Briefly, the total cellular RNA was extracted from cells using the Trizol reagent, following the manufacturer's instructions, and was retrotranscribed as previously described [15]. Next the following primer pair and probe for MxA were added to the universal PCR master mix (Applied Biosystems) at 300 and 100 nM, respectively, in a final volume of 50 mL. (forward primer, 5'-CTGCCTGGCAGAAAACTTACC-3'; reverse primer, 5'-CTCTGTTATTCTCTGGTGAGTCTCCTT-3'; probe, 5'CATCACACATATCTGTAAATCTCTGCCCCTGTTAGA-3'). Co-amplification of the beta-glucuronidase gene (Assay-On-Demand, Hs99999908_mL, Applied Biosystems) was used to normalize the amount of total RNA present. The relative amount of each transcript, normalized to beta-glucuronidase mRNA, was calculated using the arithmetic formula (2 - ΔCt) or (2 - ΔΔCt) according to the supplier's guidelines (Applied Biosystems).

### Taqman quantitative RT-PCR for microRNAs

MicroRNAs (miR-1, miR-30, miR-128, miR-196, miR-296) in PBMCs collected from patients with CHC and healthy individuals were quantified by a real time 5' exonuclease RT-PCR Taqman assay.

All primer and probes of each miRNA investigated were present in the TaqMan microRNA assays purchased from Applied Biosystems.

MiRNAs were extracted from the cells using the mirVana miRNA Isolation Kit (Ambion, Austin, TX), according to the manufacturer's protocol. Applied Biosystems TaqMan MicroRNA Reverse Transcription Kit (Applied Biosystems, Monza, Italy) was used (following the manufacturer's protocol) for reverse transcription (RT) of extracted total miRNAs. Each RT reaction contained 5 ng of extracted total miRNA, 3 μL of TaqMan MicroRNA assays, 1.50 μL of RT10x buffer, 0.25 mM each of dNTPs, 3.33 U/μL Multiscribe reverse transcriptase and 0.25 U/μL RNase inhibitor. The 15 μL reactions were incubated in a Biometra T3 Thermocycler (MMedical, Italy) in a 96-well plate for 30 minutes at 16°C, 30 minutes at 42°C, 5 minutes at 85°C, and then held at 4°C. For the real-time PCR step, amplification was carried out using TaqMan MicroRNA assays (Applied Biosystems) on the Applied Biosystems 7000 Real-Time PCR system. The 20 μL reaction included 1.33 μL RT product, 10 μL of TaqMan Universal PCR Master Mix with no UNG and 1 μL of TaqMan MicroRNA assays. The reactions were incubated in a 96-well optical plate at 95°C for 10 minutes, following by 40 cycles of 95°C for 15 s and 60°C for 1 minute. Real-time PCRs for each miRNA were run in triplicate. The relative expression levels of each miRNA were measured using the constitutively expressed RNU6B as endogenous control The expression of each miRNA relative to RNU6B was determined using the arithmetic formula (2 - ΔCt) or (2 - ΔΔCt) according to the supplier's guidelines (Applied Biosystems).

### Statistical analysis

All results are expressed as the mean ± standard deviation (median). The coefficient of variation (CV) was used to measure the interpatient variability in blood concentrations of miRNAs and MxA. Levels of miRNAs and MxA observed in PBMCs from three healthy individuals before and after the stimulation *in vitro *with IFN alpha were compared using a T-test as suggested by Bland and Altman [16]. Differences between patients with CHC and healthy individuals, and between patient groups, in terms of blood concentrations in miRNAs and MxA, were compared using the Wilcoxon test. The same test was used to assess differences between miRNAs and MxA expression levels in patients with CHC. A Spearmen rho coefficient was calculated to assess the correlation between pre-dose and HCV viral load, ALT status. Significance was fixed at the 5% level. Analysis was performed using spss version 13.0 for Windows.

## Results

### Baseline and in vitro IFN alpha induced expression of microRNAs in PBMCs collected from healthy individuals

As there are no published reports about expression profiles of miR-1, miR-30, miR-128, miR-196, and miR-296 in PBMCs from normal individuals, our first investigation was of their expression in PBMCs from healthy volunteers using real-time quantitative RT-PCR. The expression of MxA in these individuals was also evaluated for control purposes.

PBMCs isolated from healthy donors were found to express all the miRNA considered with varying expression levels, depending on the examined miRNA type. Specifically, the baseline miRNA values in PBMCs that were determined using the equation (2 - ΔCt), according to the supplier's guidelines, ranged between 0.30 and 128.96. MxA-mRNA levels were also found in PBMCs from all healthy donors (Table 2).

**Table 2:** Baseline expression of microRNAs and MxA-mRNA in healthy controls and in patients with chronic hepatitis C (CHC)

	Healthy controls* n = 10	Patients with CHC* n = 12	Mean ratio CHC/healthy controls
miR-1	0.30 **± **1.43 (0.36)	2.82 **± **5.09 (0.10)	9.4
miR-30	128.96 **± **93.99 (128.61)	255.57 **± **466.62 (27.41)	1.98
miR-128	1.39 **± **3.31 (0.05)	19.54 **± **36.15 (0.03)	14.05
miR-196	1.85 **± **3.21 (0.73)	0.81 **± **1.52 (0.04)	0.43
miR-296	3.39 **± **7.12 (0.46)	12.94 **± **40.96 (0.31)	3.81
MxA**	2.45 **± **1.05 (0.02)	9.42 **± **10.64 (7.09)	3.84

* Data are expressed as mean ± standard deviation (median).

**Figure 1:**
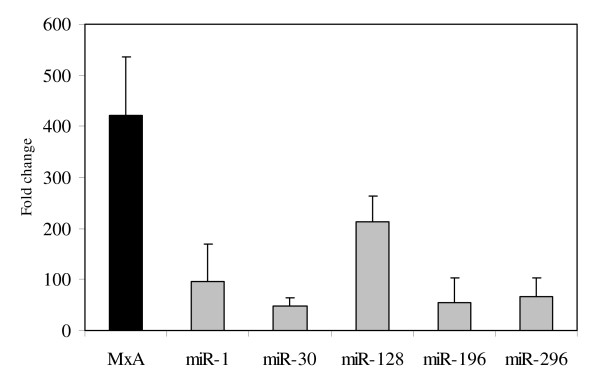
**Interferon (IFN) induced expression of microRNAs (miR-1, miR-30, miR-128, miR-196, miR-296) in peripheral blood mononuclear cells collected from three healthy individuals after in-vitro treatment with IFN alpha (100 international unit (IU)/ml)**. Expression of MxA-mRNA was also evaluated. Significant increases, relative to baseline, after in vitro IFN treatment were observed for miR-1, miR-30, miR-128, miR-196, miR-296 and MxA (p < 0.05 using student's T-test).

We then examined whether leukocyte IFN alpha could stimulate in-vitro expression of the miRNAs listed above as previously reported for IFN beta. PBMCs, freshly isolated from three healthy individuals, were treated *in vitro *with IFN alpha at 100 IU/ml (leukocyte, Alfaferone), and levels of miRNA and MxA-mRNA were measured 20 hours later by quantitative real-time RT-PCR. Again, levels of MxA transcripts were measured as positive controls for IFN action. The results showed that IFN alpha in-vitro treatment of PBMCs leads to a transcriptional induction of all miRNAs investigated as well as MxA-mRNA (Figure 1). In particular, of the miRNAs detected 20 hours post treatment, miR-1 and miR-128 had increased the most relative to untreated PBMCs, whereas miRNA-30 had increased the least.

### Expression of microRNAs in PBMCs collected from patients with CHC before and after the first injection of IFN alpha

Having established that a baseline expression of miR-1, miR-30, miR-128, miR-196 and miR-296 could be recorded in PBMCs collected from healthy donors before and after in-vitro IFN alpha treatment, we decided to analyse the expression of the same miRNAs, as well as MxA, in 12 patients with CHC, of whom 7 were classified as responders and five as non-responders to Peg-IFN alpha plus ribavirin therapy. Blood samples were collected before and 12 hours after the first Peg-IFN alpha administration.

Patients with CHC expressed baseline levels of all examined miRNAs but the levels were highly variable (CV > 100%). Importantly, the levels of expression of miRNAs were different for patients with CHC compared with healthy controls. There were higher levels of almost all miRNAs in patients with CHC compared with healthy individuals with the exception of miR-196 (Table 2). The differences did not reach statistical significance, probably because of the low number of patients and the wide variability in miRNA expression observed in them. As expected, the same trend was also observed for MxA.

**Figure 2:**
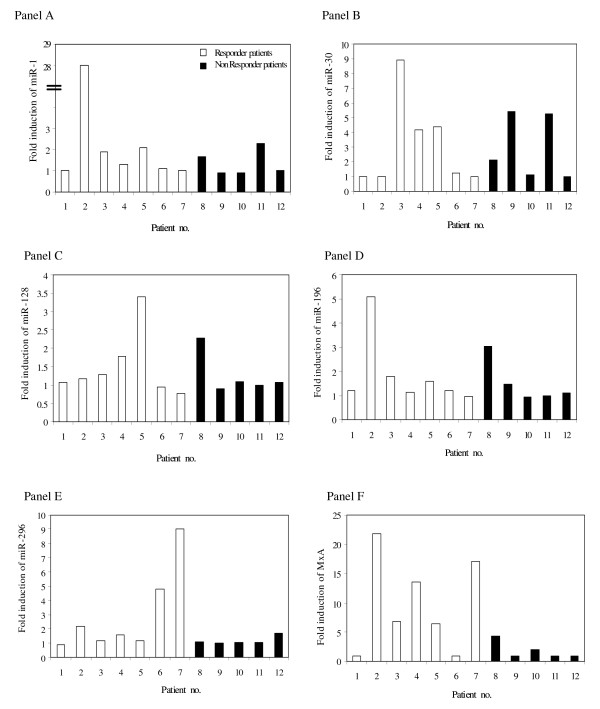
**Fold induction of microRNAs (miR)-1 (Panel A), miR-30 (Panel B), miR-128 (Panel C), miR-196 (Panel D), miR-296 (Panel E) and MxA-mRNA (Panel F) in peripheral blood mononuclear cells collected from all single patients with chronic hepatitis C after interferon treatment**.

We then examined the expression of IFN-induced miRNAs and MxA in patients with CHC after the first injection of IFN alpha. The results are shown in Figure 2. It can be seen that 12 hours after IFN alpha administration, a greater than 1.5-fold increase in MxA was recorded in 58% of patients with CHC, whereas IFN induction of miRNAs varied between 25% and 50%, depending on the type of miRNA examined. The greatest increase in miRNA and MxA levels after IFN injection were observed independently in patients no. 2 (miR-1, miR-196 and MxA), no. 3 (miR-30), no. 5 (miR-128) and no.7 (miR-296).

**Figure 3:**
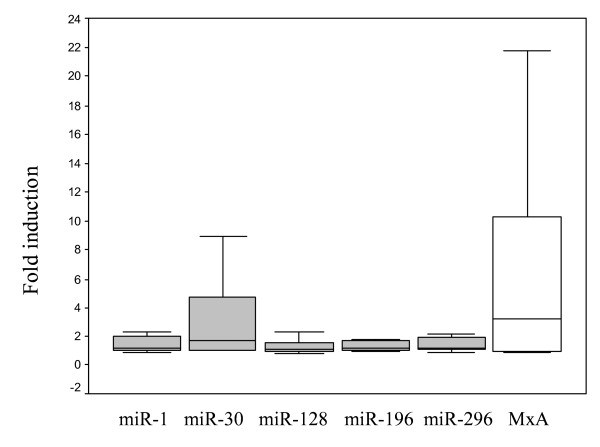
**Fold induction of microRNAs (miR)-1, miR-30, miR-128, miR-196, miR-296 and MxA-mRNA in peripheral blood mononuclear cells collected from patients with chronic hepatitis C after the first injection of interferon**. Significant increases, relative to baseline, after IFN treatment were observed for miR-1, miR-30, miR-296 and MxA-mRNA (p < 0.05 using Wilcoxon test).

In addition, different increases after IFN treatment relative to baseline were observed for miR-1, miR-30, miR-296 and MxA (p < 0.05) (Figure 3).

The baseline levels of miRNAs were also analysed to determine whether the expression of these molecules could be associated with the clinical outcome of IFN therapy. The analyses showed that baseline levels of miRNAs were not significantly different between responders and non-responders (Table 3). However, a trend toward higher baseline expression of miR-296 was observed in non-responder compared with responder patients. In contrast, miR-128 and miR-196 tended to be higher in responders than in non-responder patients.

**Table 3:** Baseline and IFN-induced expression of microRNAs and MxA-mRNA in patients with chronic hepatitis C according to the response to antiviral therapy (Peg-interferon (IFN) and ribavirin)

	Baseline expression*		Fold IFN induction*	
	**Responders**	**Non-responders**	**Responders**	**Non-responders**

miR-1	2.61 ± 4.03 (0.52)	3.12 ± 6.84 (0.07)	5.26 ± 10.23 (1.29)	1.35 ± 0.60 (1.00)
miR-30	222.10 ± 446.32 (19.61)	302.42 ± 543.38 (31.52)	3.09 ± 2.97 (1.20)	2.99 ± 2.21 (2.12)
miR-128	31.25 ± 42.77 (0.04)	0.01 ± 0.01 (0.007)	1.48 ± 0.90 (1.15)	1.27 ± 0.57 (1.08)
miR-196	1.19 ± 1.78 (0.05)	0.05 ± 0.03 (0.04)	1.85 ± 1.46 (1.21)	1.51 ± 0.87 (1.12)
miR-296	0.77 ± 1.01 (0.31)	34.23 ± 68.12 (0.21)	2.96 ± 2.97 (1.56)	1.18 ± 0.29 (1.07)
MxA	9.96 ± 11.17 (7.16)	8.67 ± 11.09 (2.99)	9.68 ± 8.02 (6.86)	1.82 ± 1.49 (0.97)

*Data are expressed as mean ± standard deviation (median).

Although we were unable to reach a definitive conclusion because there were too few patients, the overall expression of miRNA induced after IFN administration was observed to be no different in responders than in non-responder patients. In contrast, and as expected [17], responder patients were characterized by a higher induction of MxA after IFN administration compared with non-responders (p = 0.07).

We also analysed the baseline expression and the level of increase of miRNAs and MxA after IFN alpha treatment in relation to the HCV genotype, ALT levels and RNA viral load, but no significant association was found (data not shown).

## Discussion

We have demonstrated for the first time that miRNAs, previously reported to be involved in IFN-mediated antiviral activity against HCV, are expressed in PBMCs collected from healthy individuals, and that their expression in such cells may be induced by IFN-alpha to varying degrees. Specifically, greater increases in miR-1 and miR-128 and a lower increase in miR-30 were recorded in IFN alpha-treated PBMCs. This is in agreement with Pedersen and co-authors, who observed a high-fold increase in miR-1 and a low-fold increase in miR-30 in experiments performed *in vitro *with Huh7 cells treated with IFN beta [4]. However, the same authors also observed that, in primary hepatocytes, miR-1 and miR-30 expression increased at the same levels after IFN beta treatment. The differences in the levels of miRNAs induced after in-vitro IFN treatment and the Pedersen study may reflect the different sensitivities of each cell type to IFN action in terms of miRNA induction, as also reported by others [18,19].

Having established that PBMCs from healthy controls expressed the above miRNAs before and after IFN alpha treatment, the study then focused on evaluating whether PBMCs collected from patients with CHC expressed baseline levels of miRNAs and how IFN administration could modulate their expression. The results showed that PBMCs from patients with CHC had a trend towards greater expressions of these miRNAs compared with healthy controls, with the exception of miR-196. These findings are new but not surprising because, in agreement with earlier studies, they could indicated greater endogenous activation of IFN-induced pathways in patients with CHC than in healthy controls [20-23].

As far as the influence of baseline expression of these miRNAs on the clinical outcome of IFN therapy in patients with CHC is concerned, slight, although not significant, differences were observed between responders and non-responders. Several studies have shown that HCV-positive patients with elevated ISGs expression tend to respond poorly to therapy compared with patients with low baseline expression [17,24-26]. The cause of these different responses to therapy is not understood. It can be speculated that patients with CHC who have elevated initial expression were refractory to further stimulation of ISGs by exogenous IFN. We observed in our previous study that there was an inverse correlation between the relative increase in IFN-induced biomarkers and their baseline levels in patients with CHC or multiple sclerosis [23]. However, in this study, no inverse correlation was found between baseline expression of miRNAs and their levels after IFN induction (data not shown). Moreover, although IFN alpha was seen to induce changes in miRNA expression in patients with CHC, and that the highest increase in each miRNA was seen only in responder patients, no significant differences were found in the expression levels of IFN-induced miRNAs between responders and non-responders, and HCV-RNA levels appeared to have no influence on the baseline expression of IFN-induced miRNAs.

It is reasonable, therefore, to speculate that IFN treatment as well as HCV infection could affect the expression of these miRNAs in the liver more than in the PBMCs. In this regard, it has recently been shown that patients with CHC who had no virological response during later IFN therapy had markedly low baseline levels of miR-122, whereas only limited changes were seen for the other miRNAs investigated [27]. However, the authors did not compare the expression of IFN-induced miRNAs in the liver and in the PBMCs collected from the same patients with CHC undergoing IFN treatment. In addition, Meier and co-authors reported recently that while IFN-alpha treatment led to the induction of type I IFN regulated genes in PBMCs, such an induction appeared not to occur in the livers of patients with hepatitis C, which suggests that the mechanism by which IFN-alpha treatment causes viral clearance might be independent of hepatic activation of type I IFN regulated genes [28]. All this indicates more clearly the complexities of the analysed phenomena and the difficulties in interpreting our data. A better understanding of the regulation of HCV-specific miRNA induction both in the liver and in PBMCs is required to shed light on these important and critical issues. Unfortunately, we consider that no firm conclusions can be drawn with regard to the relationship between baseline or IFN-induced miRNA expression and the clinical outcome of IFN alpha therapy in patients with CHC.

The limitations of this study included the relatively small number of patients with CHC on whom miRNA analyses were performed. Indeed, although we found differences in IFN-induced miRNA expression between healthy controls and patients with CHC, as well as between responder and non-responder, the results often did not reach statistical significance thus limiting the potential application of these data. Furthermore, the size of samples was just enough to perform all the experiments shown in the present study, and the expression of other IFN-induced pathways that would have been of interest could not be evaluated. Another possible source of bias derives from the fact that, for ethical reasons, we collected only one blood sample after the IFN alpha therapy began. We consider that a more extensive analysis of IFN-induced miRNAs, including blood samples collected from CHC patients at multiple time points after therapy started, would allow the provision of a more careful analysis of the phenomenon, possibly by exploring the intriguing results we have obtained. Indeed, since it has been demonstrated that there is a significant induction of IFN-induced genes between 12 and 24 hours after IFN administration [9-14,23,29], it is possible that taking samples earlier would provide additional results, as suggested by Sarasin-filipowicz and co-authors [30].

This study extends previous investigations into the activation of the IFN system in patients with CHC and, specifically, the ability of type I IFN to regulate miRNA expression [4,27]. In particular, we have demonstrated that IFN alpha in-vitro treatment of PBMCs leads to transcriptional induction of miRNAs. We have also demonstrated, for the first time to our knowledge, that miRNA expression could be measured in PBMCs collected from patients with CHC both before and after IFN alpha administration. Larger longitudinal studies are required to gain a better understanding of the activation of IFN-induced miRNAs in patients affected by CHC.

## Competing interests

The authors declare that they have no competing interests.

## Authors' contributions

CS was responsible for design of the study, execution of the Taqman experiments, performing data analysis and writing the manuscript; PZ was responsible for performing selection of patients with CHC and analysing of clinical data; JV was responsible for performing selection of patients with CHC and analysis of clinical data; CS was responsible for executing the TaqMan experiments and analysis the HCV-positive patient data, DR was responsible for performing selection of patients with CHC and analysing of clinical data; GT was responsible for analysis of the data and revising of the manuscript; ER was responsible for helping into the design of study and analysis of the miRNAs data; EP was responsible for selection of patients with CHC, analysis of clinical data, revising of the manuscript and grants owner; GA was responsible for helping into the design of the study, writing of the manuscript, and grants owner. All authors read and approved the final manuscript.
